# Regulating effect of virtual reality restorative environment on prefrontal cortex dysfunction after night shifts in medical staff: an fNIRS study protocol for a randomized controlled trial in Dalian, China

**DOI:** 10.1186/s13063-023-07227-x

**Published:** 2023-05-23

**Authors:** Xiaofeng Huang, Cuiyan Song, Yingjun Jiang, Zhanhua Liang, Xiaotong Qu, Shaoyan Fu

**Affiliations:** Department of Neurology, the First Affiliated Hospital of Dalian Medical University, Dalian, Liaoning Province, China

**Keywords:** Virtual reality, Functional near-infrared spectroscopy, Medical staff, Night shift, Stress, Work overload

## Abstract

**Background:**

Night shift work-related disturbed biological rhythm and insufficient sleep affect the functioning of brain activity and thus impair cognitive performance and mood state, which potentially leads to negative and even devastating results for both individuals and patients. A virtual reality (VR)-based restorative environment has shown to be an effective new technique to reduce stress and improve cognitive performance, but little is known about its mechanism of improving neuronal activity and connectivity.

**Methods:**

This is a randomized, controlled, single-center clinical trial. A total of 140 medical staff will be enrolled and randomized in a 1:1 allocation to either the VR immersion group (intervention group) or the control group. In the morning after the night shift, the participants in the intervention group will watch 360° panoramic videos of immersive VR natural restorative environments for 10 min, while the participants in the control group will just rest for 10 min. Assessments of abbreviated Profile of Mood States Questionnaire (POMS) and verbal fluency task (VFT) performances, as well as oxygenated hemoglobin (oxy-Hb) and deoxygenated hemoglobin (deoxy-Hb) and total hemoglobin concentration acquired by functional near-infrared spectroscopy (fNIRS) will be performed at baseline (day work), the morning after night shift but before the intervention (previous) and after intervention (post). Data collected after a night shift will be compared to baseline performance as well as between the two groups.

**Discussion:**

This trial will investigate the effects of the night shift and VR-based restorative environment intervention on mood, cognitive performance, and neuronal activity and connectivity. A positive result in this trial could encourage hospitals to apply VR technology to reduce physical and mental dysfunction during of night shifts among medical staff in every department. Furthermore, the findings from this study will contribute to understanding the underlying neuromodulation mechanisms of how restorative environments influence mood and cognition.

**Trial registration:**

Chinese Clinical Trial Registry ChiCTR2200064769. Registered on 17 October 2022.

## Administrative information

**Table:** 

Title {1}	Regulating effect of virtual reality restorative environment on prefrontal cortex dysfunction after night shifts in medical staff: an fNIRS study protocol for randomized controlled trial in Dalian, China
Trial registration {2a and 2b}.	Protocol Record number is PJ-KS-KY-2022-334. WHO Trial Registration DataSet is not available. Trial was registered at www. chictr.org
Protocol version {3}	Version 1.1, 20 September 2022 with the Identifier of ChiCTR2200064769
Funding {4}	This study is supported by the Medical Science Research Program of Dalian (Grant No. 1812009)
Author details {5a}	^1^ Department of Neurology, the First Affiliated Hospital of Dalian Medical University, Dalian, Liaoning Province, China
Name and contact information for the trial sponsor {5b}	First Affiliated Hospital of the Dalian Medical University 222 Zhongshan Road, Dalian, Liaoning, China, 116011 E-mail: dyyyirb@163.com Phone: +86 0411-83635963
Role of sponsor {5c}	Sponsors did not have any role in designing the study protocol

## Introduction

### Background and rationale {6a}

Medical staff often work night shifts to ensure the health of patients. Since night shift work is associated with a disturbed biological rhythm and insufficient sleep, this often leads to psychosomatic stress, such as depression, cognitive impairment, burnout, cardiac autonomic dysregulation, and increased risk of work errors [1–5]. The COVID-19 pandemic has exacerbated stress and burnout among medical staff. Multiple studies have confirmed that night shift work has a passive effect on advanced cognitive function, including working memory capacity, speed of processing information, perceptual reasoning, and cognitive flexibility [2]. The prefrontal lobe is known to play an important role in emotional control and higher cognitive functions. Functional neuroimaging studies have shown that night duty leads to reduced brain activity and decreased brain functional connectivity density of the frontal cortex [3, 6, 7]. Therefore, an increasing number of studies have focused on the damaging effects of night shifts on medical staff and the development of effective interventions that can relieve stress, which would be valuable in enhancing patient safety and protecting the health of medical staff.

Restorative environments (mainly natural environments) have been beneficial for restoring attention, reducing stress, and improving physical and mental health, and could potentially meet the physical and mental needs of medical staff [8, 9]. Recently, VR technology has shown great potential in the field of mental health, and the combination of restorative environments and VR technology has been effective for emotional and cognitive recovery [10–12]. An EEG study showed that the VR restorative scene experience could activate the frontal lobe, suggesting an important mechanism that could be conducive to cognitive and emotional recovery [12]. Furthermore, VR technology had been used to decrease the physical and mental workload of anesthesiologists on night shifts and reduce the stress response and cognitive load of inpatient oncology nurses [13, 14]. Therefore, the VR restorative environment may be an effective intervention measure to reduce night shift stress, which would be of great significance in improving medical safety and protecting the health of medical staff. However, there are no studies yet that have investigated the neural mechanism of the regulatory effect of the VR restorative environment on frontal lobe activity after night shift work.

Previously, fNIRS studies have shown decreased prefrontal cortex (PFC) activity in night duty physicians [6]. In addition, night shift sleep deprivation impairs performance on cognitive tasks, including VFT, which are thought to rely on PFC involvement and are less activated by cognitive tasks in sleep deprivation (night shift) than in rested subjects (day shift) [7]. However, how this abnormal activation can be regulated by VR has not been reported. This protocol describes the implementation of watching 10 min of a VR restorative environment among medical staff after a night shift, and fNIRS is used to measure the changes in frontal lobe activity before and after the intervention.

### Objectives {7}

This trial will investigate the effects of the night shift and VR-based restorative environment intervention on mood, cognitive performance, neuronal activity, and brain functional connectivity. A positive result in this trial could encourage hospitals to apply VR technology to reduce physical and mental dysfunction during night shifts among medical staff in every department. Furthermore, the findings from this study could contribute to understanding the underlying neuromodulation mechanisms of how restorative environments influence mood and cognition.

### Trial design {8}

**Fig. 1:**
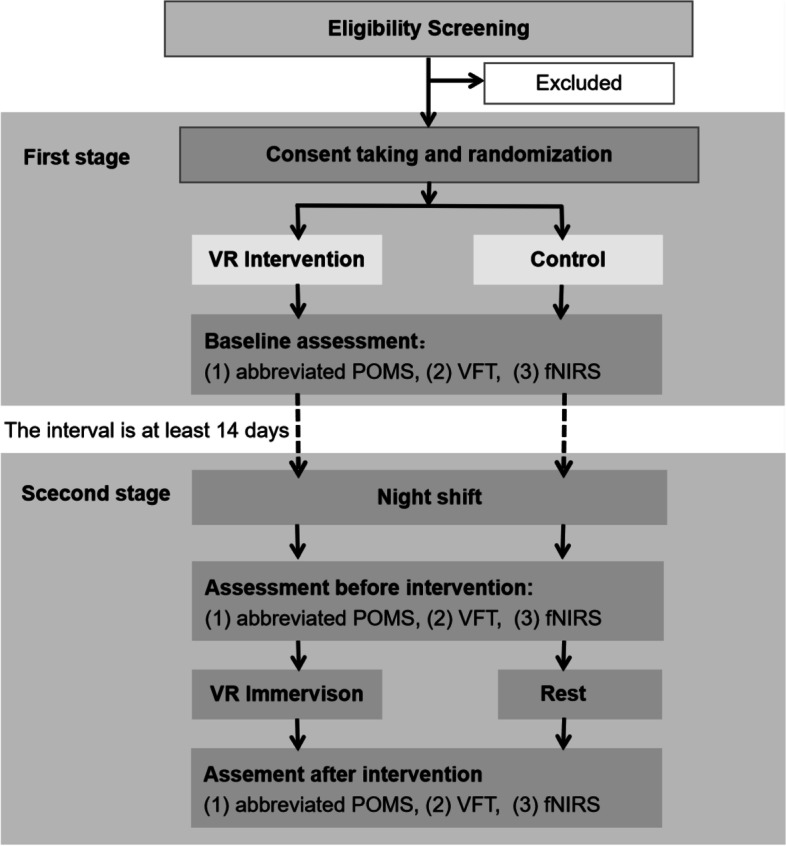
Expected trial plan. Not included in the diagram are excluded participants who may stop participation at any time for various reasons. The intervention is 10 min.

The study is a parallel two-group randomized controlled trial comparing the mood, cognitive performance, and brain activation of medical staff who undergo and do not undergo VR immersion after a night shift. In addition, data collected from a day shift are assessed and serve as a normal baseline. A total of 140 volunteers (including physicians and nurses) will be randomized in a 1:1 allocation to the intervention group and the control group by the block randomization method. fNIRS and abbreviated POMS assessments will be used at three time points collected throughout the study to assess how night shift and VR restorative environment immersion affect frontal cortex activity and mood in physicians and nurses [15, 16]. The collection time points are as follows: the first is collected during the day shift which is at least 3 days after the last night shift, and the last two are collected in the morning after the night shift (at 8:00–9:00 am) before and after VR intervention/rest. The interval between day shift and night shift assessments is at least 14 days. The intervention group will undergo immersion relaxation by watching their favorite VR restorative environment video for 10 min at 8:30 am after the night shift, while the control group will rest quietly for 10 min awake. The timeline design of the study is presented in Fig. 1.

The primary outcomes of the study will be the different cerebral oxygenation levels assessed during VFT performances using fNIRS, to evaluate the alterations in regional activity and functional brain network connectivity in the PFC. Secondary outcomes will be the abbreviated POMS scores at all the recorded time points [16].

## Methods: participants, interventions, and outcomes

### Study setting {9}

The study will be conducted in the First Affiliated Hospital of Dalian Medical University, a large comprehensive hospital in Dalian, China. This is a teaching hospital, including more than 90 nurses and more than 100 physicians aged from 20 to 45 in the Department of Neurology. The night shift is from 17:00 to 8:00 of the next day.

### Eligibility criteria {10}

Potential participants will be recruited from the Neurology Department of the First Affiliated Hospital of Dalian Medical University. Only individuals who meet the inclusion criteria will be invited to participate in the study.

Eligibility criteria include the following:

1. Aged 20–45 years old
2. Regular shift work for more than 1 year
3. Right-handed

Exclusion criteria include the following:

1. History of neurological diseases, head trauma, or surgery
2. Using sedatives or stimulants
3. Other reasons for ineligibility

### Who will take informed consent? {26a}

A list of eligible participants includes medical staff from the First Affiliated Hospital of Dalian Medical University. ZL is responsible for the recruitment and screening of eligible participants. XH will complete study enrollment along with obtaining informed consent. Solicitation to eligible participants who meet the established study criteria will be made face to face. Participants will be invited to review the study details, address any questions, and sign the consent form. No later than 1 week after the consent form is signed, baseline measurements will be taken. Consent will be collected using a short form provided in Simplified Chinese. The form includes a description of the study, reasonably foreseeable risks or discomfort to the participant, and the rights of the participant, including withdrawal of participation at any time. The form may be signed by hand or via electronic signature. A copy of the form will be provided (paper and/or electronically) to the participant upon signage for the participant to keep as a record.

### Additional consent provisions for collection and use of participant data and biological specimens {26b}

On the consent form, participants are asked if their data can be used even if they choose to withdraw from the trial. Participants are also asked for permission of the research team to share relevant data with personnel from regulatory authorities, where relevant. The trial does not involve the collection of biological specimens.

## Interventions

### Explanation for the choice of comparators {6b}

To assess the damaging effects of the night shift, the brain activity, VFT performance, and mood state outcomes of the day shift will be compared with those after the night shift. To assess the beneficial effects of VR-based restorative environment intervention on mood, cognitive performance, and brain activity, those outcomes will be compared among pre- and post-VR intervention and simple relaxation.

### Intervention description {11a}

The intervention time point is approximately 8:30 am after the night shift. All participants will complete the fNIRS and abbreviated POMS first, which takes approximately 20 min. Participants in the intervention group will recline comfortably on a sofa, wearing a VR glass loaded with an intelligent VR psychosomatic interactive training system (Ruixin, Ruihai Yinuo Intelligent Technology Co., Ltd., China) and watch 360° panoramic videos of the immersive VR natural restorative environment selected according to their preferences to relax themselves in the resting room. The intervention time will be 10 min. After the intervention, all participants will complete the fNIRS and abbreviated POMS again. Participants in the control group also recline comfortably on a sofa but only rest for 10 min without VR immersion. After relaxation, all participants will complete the fNIRS and abbreviated POMS again.

### Criteria for discontinuing or modifying allocated interventions {11b}

Participants may quit at any time due to various reasons including failure to meet inclusion criteria or inability to continue VR intervention.

### Strategies to improve adherence to interventions {11c}

Not applicable because it is a one-time intervention.

### Relevant concomitant care permitted or prohibited during the trial {11d}

Not applicable because all participants are healthy.

### Provisions for posttrial care {30}

Not applicable to study; no harm is anticipated from this study as the equipment used poses little risks.

### Outcomes {12}

The primary outcomes are the oxy-Hb and deoxy-Hb concentration of brain cortex acquired by fNIRS and VFT performances at three assessment time points (baseline, after the night shift before VR immersion, after VR immersion), to evaluate the alterations of in regional brain activity and functional brain network connectivity in the PFC. The secondary outcomes will be the abbreviated POMS scores at all the recorded time points.

### Participant timeline {13}

Figure 1 graphically shows the participant timeline.

**Fig. 2:**
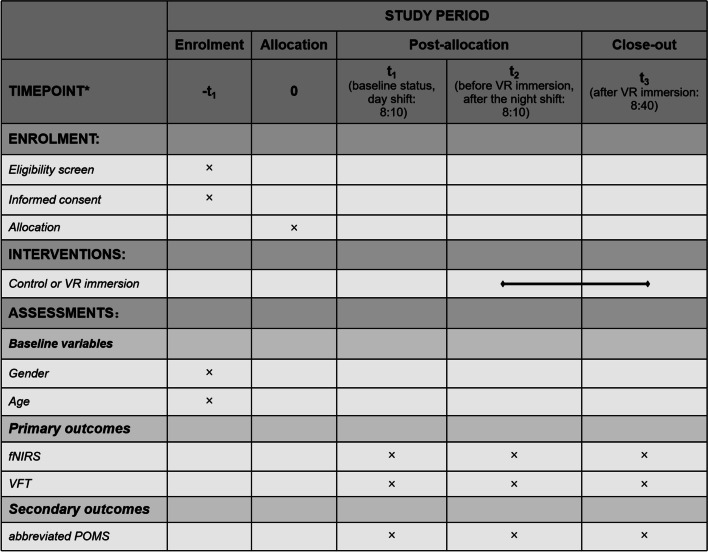
Standard Protocol Items: Recommendation for Interventional Trials (SPIRIT). *VR* Virtual reality, *VFT* Verbal fluency test, *fNIRS* Functional near-infrared spectroscopy, *POMS* Profile of Mood States Questionnaire.

Figure 2 illustrates the standard protocol.

### Sample size {14}

In this protocol, a total of 140 participants (70 participants in two groups) will be recruited over an 18-month period. We aim to recruit an even distribution of age and gender to maximize statistical significance. The primary outcomes are the oxy-Hb and deoxy-Hb concentrations of the brain cortex acquired by fNIRS at baseline, after the night shift before VR immersion, and after VR immersion (or after rest). We assumed that the VR intervention group would show better recovery of PFC function than the non-intervention group. Because there are no pilot studies or previous studies, the sample size was calculated using G*Power software (downloadable from: https://download.cnet.com/G-Power/3000-2054_4-10647044.html), with 80% power, 2-sided 5% significance level, and a moderate effect size *d*=0.5. We assume that the mean of the two groups are the same. This sample size of 140 participants also allows for 10% loss to follow-up. Durán-Gómez N et al.’s study was used to estimate the baseline averages of oxy-Hb changes[7]. However, there are notable inherent differences between this protocol and Durán-Gómez N et al.’s study because the latter only described oxy-Hb changes before and after a night shift without other intervention [7].

### Recruitment {15}

Potential participants will be recruited from the First Affiliated Hospital of Dalian Medical University. Recruitment will be managed by ZL. We will announce recruitment via WeChat, posters, and word of mouth. In this protocol, the recruitment period will be an 18-month period. According to our preliminary intention-to-enroll survey, we estimate a recruitment rate of at least 90%. We will initially recruit participants in the Department of Neurology, and if we do not recruit enough participants, we will expand to other departments of the hospital and may extend the recruitment period as appropriate.

## Assignment of interventions: allocation

### Sequence generation {16a}

Following informed consent, participants will be randomized in a 1:1 allocation to a sequence of either immersion relaxation using VR or just taking a rest without immersion VR, based on a computer-generated random number that is concealed via a sealed envelope. One of the researchers will conduct this step. The allocation will not be disclosed to the other researchers who conduct the trial until the participants are enrolled and assigned. In addition, researchers who perform the statistical analyses will be blinded to the group allocation.

### Concealment mechanism {16b}

Once the consent form is received, a computer-generated random number from 1 to 140 will be assigned to that participant. Each number from 1 to 140 will be assigned a random group type: VR intervention or control. For the numbers randomly allotted for participants, the computer-generated random number assignment will be set such that it results in a 1:1 ratio, stratified by gender. The participant’s number will become the identifier for the participant’s collected data for data analysis. Once all participants have been enrolled and assigned, the list of which participants were distributed among the groups will be kept in an opaque, sealed envelope. Upon completion of consent, randomization, and baseline data collection, the participant will receive an opaque and sealed envelope associated with their randomized number. The researchers responsible for data collection and the analysis of outcomes will be blinded until after the completion of data analysis.

### Implementation {16c}

ZL will generate the allocation sequence and prepare the opaque envelopes with further instructions. XH will enroll and allocate participants.

## Assignment of interventions: blinding

### Who will be blinded {17a}

The researchers responsible for data collection and the analysis of outcomes will be blinded until after the completion of data analysis because they will neither be involved in the assignment of subjects nor the implementation of the interventions.

### Procedure for unblinding if needed {17b}

Not applicable to the study; the design is open label with outcome assessors and data analysts being blinded so unblinding will not occur.

## Data collection and management

### Plans for assessment and collection of outcomes {18a}

Data will be collected from participants of two groups at three time points: baseline status (08:10 am, T1), before VR immersion (08:10, T2), and after VR immersion (08:40, T3) of the next day.

#### fNIRS

**Fig. 3:**
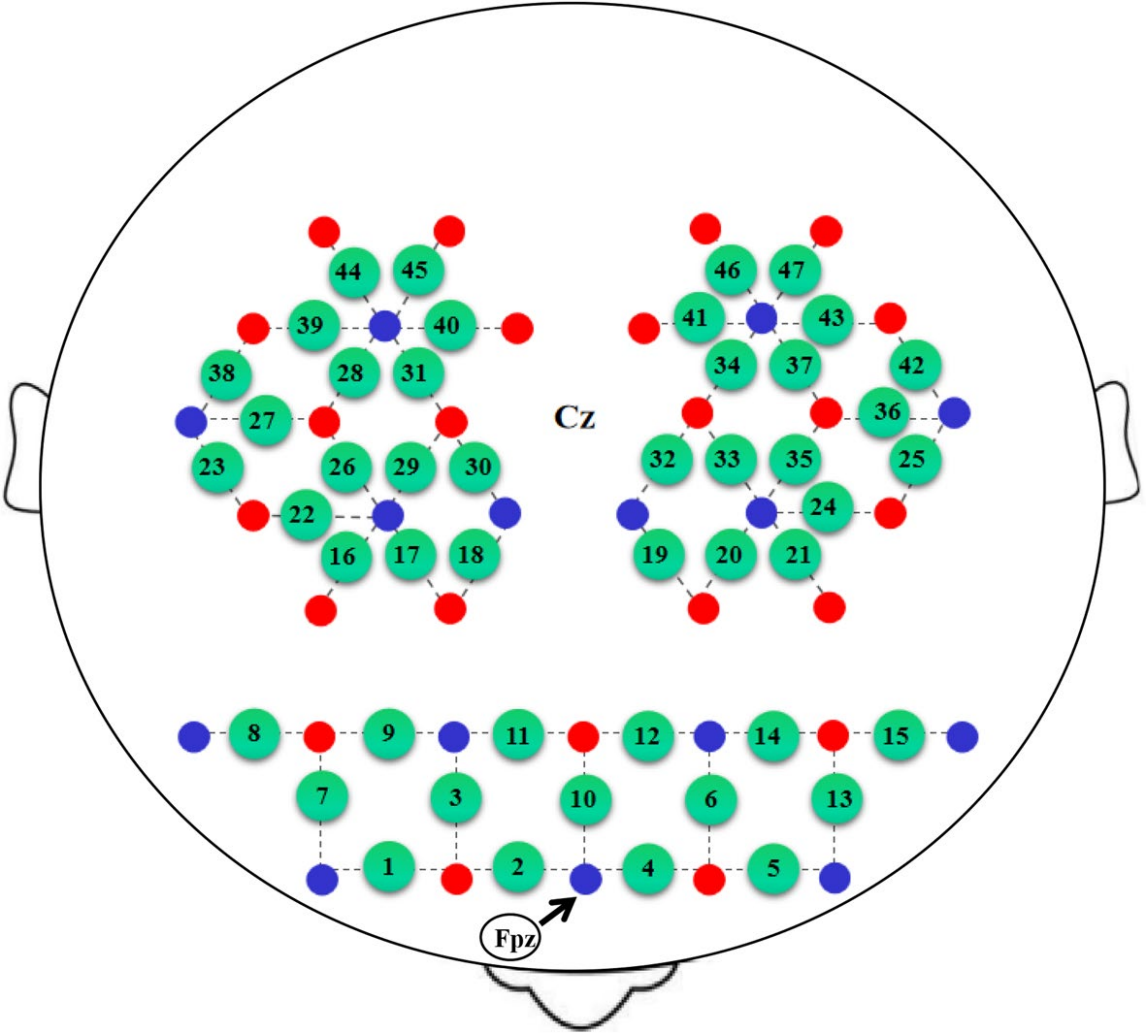
Arrangement of the fNIRS probe array. The red and blue dots represent the sources and detectors, respectively. Green dots with digits represent the positions of the measurement channels.

The fNIRS assessment will be employed to examine the cortical neural activity changes from the night shift and VR intervention. A multichannel near-infrared brain function imaging device (NirSmart, Danyang Huichuang Medical Equipment Co., Ltd., China) will be used to record changes of oxy-Hb and deoxy-Hb, and the emission light sources will be 730 nm and 850 nm, respectively. There are 23 emitters and 15 detectors to form 47 effective channels. The distance between channels is 3 cm, and the sampling rate of all channels is ≥11 Hz. The probe set will be placed on the scalp corresponding to the PFC, premotor and supplementary motor cortex, motor cortex, and sensory cortex, according to the international 10–20 system for electroencephalogram. The Cz and Fz positions from the 10–20 system are first identified. Detector D2 corresponds to the Fpz point. The midpoint of the connecting line between emitter S13 and emitter S14 corresponds to the Cz point (Fig. 3).

All participants will be told to avoid medications that may affect brain activity for at least 24 h prior to the assessment. Prior to the assessment, the participants will be read instructions and perform a training module to ensure that they understand the study and know what to expect. During the fNIRS assessment, the participants will be seated, comfortably and relaxed, in front of a computer monitor in a warm, quiet, and slightly dark room. Participants will be guided to relax and to restrict their motions during the experimental paradigm.

#### VFT task

Studies have shown that fNIRS is sensitive enough to also detect small changes during VFT [17]. The VFT has been widely used to investigate impaired PFC activation or decreased cognitive performance [18, 19]. In this protocol, VFT will be employed to test cognitive functions while assessing PFC hemodynamics by fNIRS. Before the test, the test process will be explained to the subjects with a unified guide. The test will be performed in four stages, 160 s in total. The first stage is a prescanning period, 10 s in total. The subject focuses on the 10 cm×10 cm cross on the computer screen 70 cm in front and waits for the test to begin. The second stage is a repeat count period, lasting for 30 s. The participants stare at the 10 cm×10 cm cross on the computer screen and repeat counts from 1 to 5. The third stage is the listing word task period, lasting for 60 s, in which three simple Chinese characters (such as Da, Tian, and Bai) are prompted by machine voice, and the subjects list words that start with the character, each lasting for 20 s. Stage 4 is a repeat count period again, lasting for 60 s, and the subjects still repeat counts from 1 to 5. The behavioral performance will be assessed by the total number of correct words generated in the third stage. In this protocol, fNIRS assessments while executing the VFT task will be conducted at 3 time points (day duty, before intervention after the end of the night shift, and after intervention). To avoid the influence of memory on the results when performing the task repeatedly, the Chinese characters provided at the 3 time points will all be different.

#### Abbreviated POMS

The abbreviated POMS includes seven mood state subscales: tension, anger, fatigue, depression, vigor, confusion, and esteem-related affect. It is a 40-item self-report measure that evaluates the current emotional mood state of participants. Participants will be asked to rate how they are currently feeling in response to each of the 40 adjectives on a 5-point Likert scale ranging from ‘Not at All’ to ‘Extremely’. The total mood distance (TMD) is calculated by [tension + depression + anger + fatigue + confusion] − [vigor + esteem-related affect], and finally adding a constant of 100. Overall, higher scores for TMD and negative mood states indicate higher negative mood. The abbreviated POMS has been revised in Chinese with reliability coefficients for the subscales ranging from 0.62 to 0.82, with a mean of 0.71 [16].

### Plans to promote participant retention and complete follow-up {18b}

Participants will be given a reminder (via e-mail and phone call) a week before to schedule the follow-up.

### Data management {19}

All participant data will be identified using the assigned trial identification number. Each trial identification number will have its own designated folders. Data collected from fNIRS and VFT tasks are conducted on a single laptop and will be automatically uploaded to their respective designated cloud storage databases immediately after each individual measurement. Abbreviated POMS assessments will be recorded digitally using a digital copy of the assessments and saved into their respective participant folders. All data collected throughout the study will be stored for at least 5 years. A master data sheet that collects all digital data and is organized by participant number and time point will be preprogrammed to conduct range checks for all data values collected.

### Confidentiality {27}

Data will be stored on the cloud server provided by the hospital which is already secured to ensure strict access. Only primary researchers who enroll participants or collect and analyze data and other principal members of the research team have access to the cloud database. Before the data are uploaded to the cloud, they will be organized based on trial identification numbers instead of group and individual names. Access to the database may be granted upon reasonable request from other researchers, study entities, or miscellaneous government-associated groups who wish to conduct prospective meta-analyses.

### Plans for collection, laboratory evaluation, and storage of biological specimens for genetic or molecular analysis in this trial/future use {33}

Not applicable to the study; no biological specimens will be collected.

## Statistical methods

### Statistical methods for primary and secondary outcomes {20a}

Data analysis will start after all recruited participants have completed their assessments. To avoid bias, the data analyst will be blinded to the data. Participants who successfully complete the study according to the intervention plan will be included in the analysis. Missing data or any data collected that does not adhere to the protocol will be eliminated from data analysis.

#### VFT performances and abbreviated POMS assessments

The VFT performances as well as neuropsychological measurements collected from the two groups will be compared using *t* tests.

#### fNIRS data

The raw fNIRS data will be bandpass filtered first and further processed to remove noise and drift. Neuroactivity in various regions of interest will be analyzed by constructing a general linear model for each subject then estimating parameters and obtaining contrast values of the oxy-Hb and deoxy-Hb signals. The contrast values will be averaged within groups and a group analysis will be performed using a *t* test. The averaged signal levels between two groups and between different assessment time points will be compared to determine the intervention effect.

### Interim analyses {21b}

Not applicable to the study; data analysis is not anticipated until the end of the study for all participants.

### Methods for additional analyses {20b}

Further statistical analysis may be explored to account for interacting factors including gender, age, and different hours of sleep during the night shift.

### Methods in analysis to handle protocol nonadherence and any statistical methods to handle missing data {20c}

Missing data and any data collected that does not adhere to the protocols will be removed from data analysis.

### Plans to give access to the full protocol, participant-level data and statistical code {31c}

The datasets of the study will be available from the corresponding author upon reasonable request.

## Oversight and monitoring

### Composition of the coordinating center and trial steering committee {5d}

The Department of Neurology, at the First Affiliated Hospital of Dalian Medical University, will be the trial coordinating center. The Steering Committee (chaired by ZL) is responsible for the final protocol, recruiting participants, assessing and reporting any serious unexpected adverse events, reviewing the progress of the study, and ensuring smooth running of the study. The Trial Manager (XH) is responsible for the trial’s master file, obtaining approval from the Ethics Committee, coordinating audits with the Ethics Committee, managing enrollment and allocation, releasing official changes of the clinical trial protocol to relevant entities and trial participants, scheduling participant assessments, and addressing any questions that participants have. CS will perform the intervention. Two researchers (XQ and SF) will perform data collection. YJ will be responsible for data analysis.

### Composition of the data monitoring committee, its role and reporting structure {21a}

Given that the hospital’s Ethics Committee and the study’s Steering Committee have determined the relatively low-risk nature of the trial, there will be no formal data monitoring committee. The data will be managed by a designated Data Manager. The Trial Manager (XH) will oversee data recording, and address any issues about the cloud storage, data management, and data access. XH will frequently check, and ensure that the data are collected accurately and reliably in accordance with the protocol.

### Adverse event reporting and harms {22}

It has been reported that a few people may experience physical discomfort, such as dizziness, when wearing VR glasses. Therefore, the trial can be stopped at any time if participants feel discomfort during the VR immersion, and the participants will be treated accordingly. The fNIRS system poses very little risk. Should an unforeseen serious adverse event occur, a case report will be written, and the trial manager will be notified within 24 h. All these events will be recorded and shared with the steering committee, ethics committee, and trial participants within the next 48 h. Participants will have the right to quit at any time for any reason during the trial.

### Frequency and plans for auditing trial conduct {23}

The Ethics Committee of the hospital conducts audits every six months to check on progress and ensure adherence to the established protocol. In the months leading up to the trial, the chief investigators will meet weekly with the research team to ensure that all procedures meet the standards of the protocol. During the data collection phase of the trial, this group will meet every month, with daily contact between the chief investigator (ZL) and the trial manager (XH) to ensure adherence to the protocol.

### Plans for communicating important protocol amendments to relevant parties (e.g., trial participants, ethical committees) {25}

Any amendment will be first discussed within the Steering Committee so that an agreement can be reached before it is reported to the Ethical Committee and Scientific Research Department of the hospital. Once approval is obtained, a formal document summarizing the protocol amendments will be shared with relevant parties, with digital copies available upon request. Any deviations from the protocol will be documented by the trial manager and signed off by the chief investigators. Then, this document will be stored with all other trial documentation. Necessary updates to the clinical trial application will be made by the Trial Manager after approval by the Steering Committee, Scientific Research Department, and Ethics Committee.

### Dissemination plans {31a}

We will prepare a summary of the outcomes of the RCT and email it to all participants who indicate on the consent form that they are interested in these results. Trial results will be published in peer-reviewed journal articles and delivered in presentations at national and international conferences. We will also deliver other presentations about the trial results to other hospitals if they wish to share them with their medical staff. Data can be shared upon reasonable request.

## Discussion

This study will be the first to evaluate the potential effectiveness of a VR restorative environment on PFC dysfunction among night shift medical staff using fNIRS measurements. Sleep is a vital physiological process, but shift work may increase the risk of cognitive impairment among night-shift medical staff over time [20]. Many night shift nurses and physicians show signs of mood disorders, burnout syndrome, and memory and attention problems [2, 21, 22]. As shift work is common and necessary in many professions, passive effects caused by overload work and disturbed biorhythm are substantial and should be addressed to prevent serious long-term complications.

Immersion within a restorative environment can prompt the recovery of attention and cognitive functioning and positively affect mood state [23–25]. However, for urban workers, especially medical staff who are exhausted after night shifts, a real restorative environment is usually not easy to obtain. VR technology is a convenient and economical way to experience restorative environments in hospitals, and studies have confirmed its benefits with regard to emotional and cognitive recovery [10–12]. However, there are few studies on the regulatory mechanism of the VR-based recovery environment on cerebral cortical activity after night shift work. Therefore, this study has the following two important goals: first, to explore the benefits of VR-based restorative environments on cognitive and emotional recovery after night shift work in hospitals, which will be helpful in promoting this technology in more hospitals for the benefit of more medical staff and even other night shift workers; more importantly, these findings will be helpful in understanding the effects of night shift work on cognitive impairment and exploring the neural mechanism of cognitive regulation by VR-based restorative environments.

We acknowledge that there are some limitations in this study, because there may be an effect of proficiency on the results as each participant performed the VFT task a total of 3 times throughout the study protocol. Therefore, we developed different Chinese characters at each of the 3 assessment time points, and we expect this effect to be equal between the intervention and control groups, particularly as the researchers are blinded to participant allocation. In addition, the study is a single-center trial with a small sample size. Therefore, these preliminary results should be confirmed by conducting a lage- scale multicenter study.

## Trial status

This is protocol version 1.1 (20 September 2022). Recruitment for the study will begin in October 2022 and continue for approximately 18 months.

## Data Availability

Data from this study are available upon reasonable request from the corresponding authors. The data are monitored by the corresponding authors and the scientific research department of the hospital.

## Abbreviations

VR: Virtual reality
POMS: Profile of Mood State
VFT: Verbal fluency task
oxy-Hb: Oxygenated hemoglobin
deoxy-Hb: Deoxygenated hemoglobin
fNIRS: Functional near-infrared spectroscopy
PFC: Prefrontal cortex
TMD: Total mood distance
